# Supplementary material for: Tracking blobs in the turbulent edge plasma of a tokamak fusion device
Source: Sci Rep. 2022 Oct 28;12:18142. doi: 10.1038/s41598-022-21671-w (PMC9616937; doi:10.1038/s41598-022-21671-w)
Supplement: Supplementary file 1 — Supplementary Information 1. [file 41598_2022_21671_MOESM1_ESM.pdf]

Supplementary Information for Tracking Blobs in the Turbulent Edge Plasma of a Tokamak Fusion Device

Woonghee Han[1,*], Randall A. Pietersen[2], Rafael Villamor-Lora[2], Matthew Beveridge[3], Nicola Offeddu[4], Theodore Golfinopoulos[1], Christian Theiler[4], James L. Terry[1], Earl S. Marmar[1], and Iddo Drori[3]

[1]MIT Plasma Science and Fusion Center, Cambridge, Massachusetts 02139, USA
[2]MIT, Civil and Environmental Engineering, Cambridge, Massachusetts 02139, USA
[3]MIT Computer Science & Artificial Intelligence Laboratory (CSAIL), Cambridge, Massachusetts 02139, USA
[4]École Polytechnique Fédérale de Lausanne (EPFL), Swiss Plasma Center (SPC), CH-1015 Lausanne, Switzerland
[*]harryhan@mit.edu

Supplementary Information

We provide additional details on the methods and results. Figure 1 illustrates Tracking-by-detection workflow, which is applied to the consecutive frames containing blob contours predicted by the model and assigns blob IDs based on closeness to the blobs in the previous frame. Figure 2 shows an example of computing the VIoU between the contours of a prediction (red) and a label (black). For the training of Mask R-CNN, we explore the hyperparameters in Table 1 by Bayesian optimization and find the optimal values shown in Table 2. The scores of the four models on training and validation synthetic data are shown in Table 3 with corresponding score metrics (EPE and VIoU). The EPE scores of Mask R-CNN are not applicable because it is not an optical flow detection model but a mask detection model.

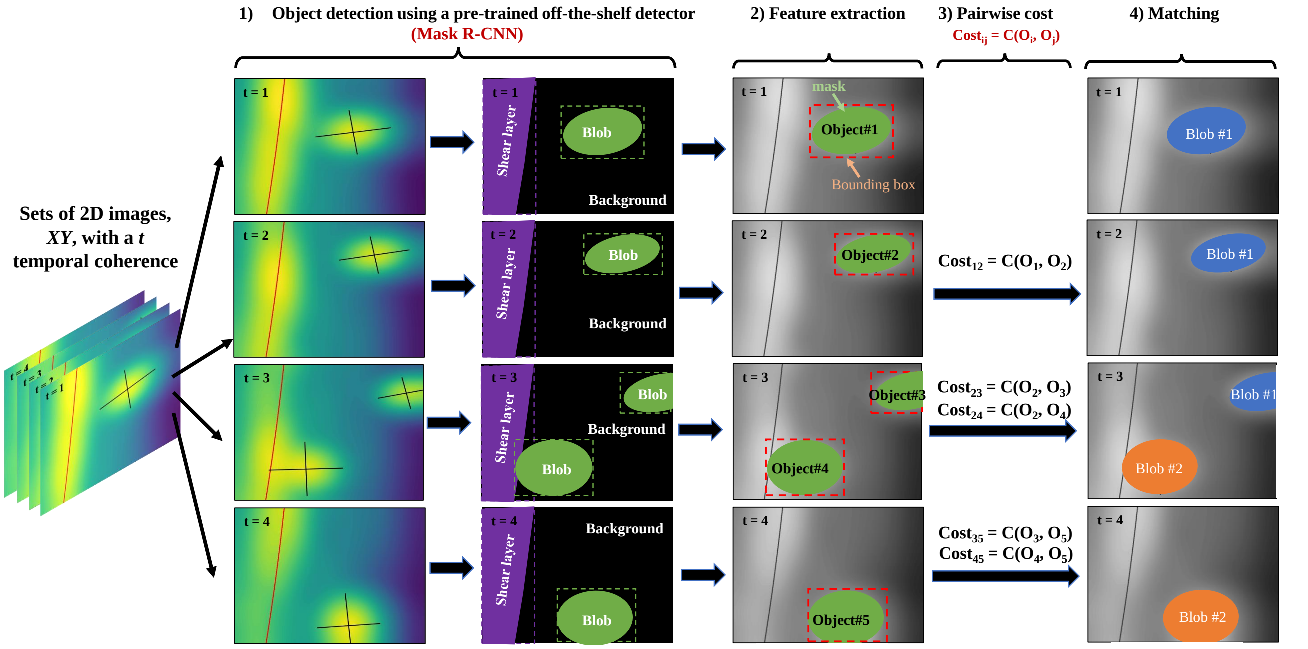

Figure 1. Tracking-by-detection consisting of four steps: (1) Object detection within each frame using a pre-trained model. Here the mask R-CNN produces a bounding box and a mask for each candidate object. (2) Extraction of features of interest (i.e., masks of blobs). (3) Computation of pairwise costs between objects in the current and previous frame. (4) Bipartite matching between objects assigning unique correspondence.

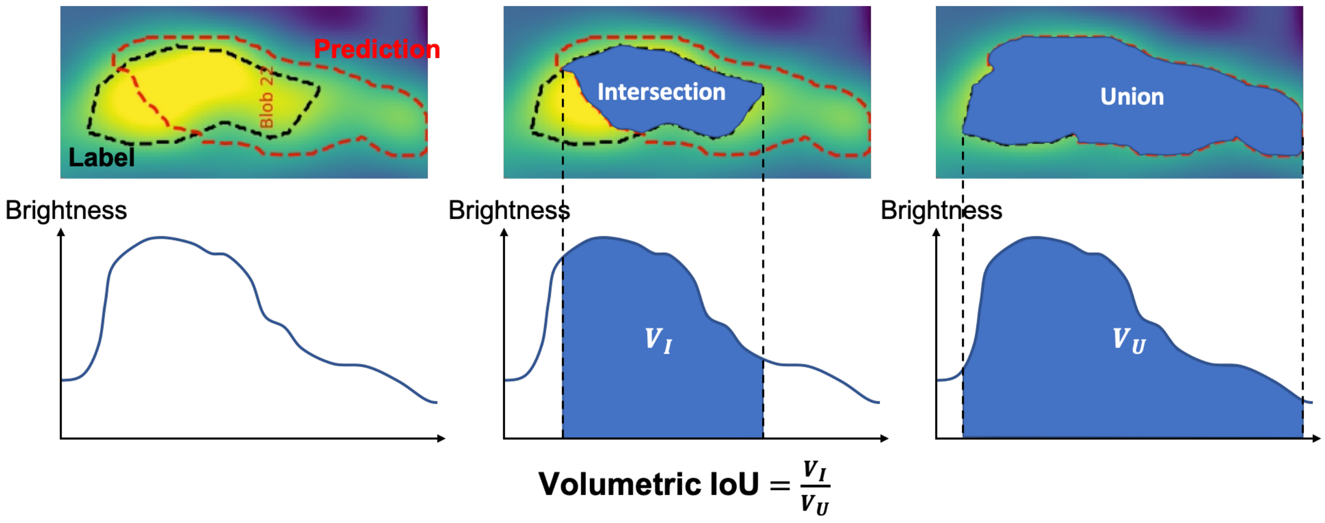

Figure 2. Volumetric Intersection over Union (VIoU) computed for a prediction on an image containing a blob (top) using the volume under the profile of the brightness (bottom).

Table 1. Exploration ranges of hyperparameters used in Bayesian optimization. BO level 1 hyperparameters: learning rate initial value, learning rate reduction period, learning rate decay factor, momentum, weight decay and number of training epochs. BO level 2 hyperparameters: probabilities for horizontal flip, scaling, translation, shearing, rotation, and dropout.

BO Lv. 1	LR initial val	LR reduction period	LR decay	Momentum	Weight decay	n_{epochs}
Exploration range	1e-6—5e-2	1—40	0.1—1.0	0.0—1.0	0.0—1e-3	2—40
BO Lv. 2	$P_{horizontalFlip}$	P_{scale}	$P_{translate}$	P_{shear}	P_{rotate}	$P_{dropout}$
Exploration range	0.0—1.0	0.0—1.0	0.0—1.0	0.0—1.0	0.0—1.0	0.0—0.5

Table 2. Optimal values of hyperparameters found from Bayesian optimization.

BO Lv. 1	LR initial val	LR reduction period	LR decay	Momentum	Weight decay	n_{epochs}
Optimal value	0.050	12	0.402	0.296	2.729e-5	28
BO Lv. 2	$P_{horizontalFlip}$	P_{scale}	$P_{translate}$	P_{shear}	P_{rotate}	$P_{dropout}$
Optimal value	0.341	0.560	0.261	0.564	0.368	1.283e-17

Table 3. Scores from training and validation for each model with corresponding score metrics, endpoint error (EPE) and volumetric IoU (VIoU). Mask R-CNN misses EPE because it is not an optical flow detection model but a mask detection model.

	Training score on synthetic data		Validation score on synthetic data	
Models\Metric	EPE	VIoU	EPE	VIoU
RAFT	0.161	0.869	0.338	0.879
GMA	0.160	0.865	0.298	0.862
Mask R-CNN	N/A	0.705	N/A	0.704
Flow Walk	0.946	0.835	0.924	0.830
